# Supplementary material for: Variance in C. elegans gut bacterial load suggests complex host-microbe dynamics
Source: PLoS Comput Biol. 2025 Jun 9;21(6):e1013110. doi: 10.1371/journal.pcbi.1013110 (PMC12180659; doi:10.1371/journal.pcbi.1013110)
Supplement: S1 Text — Fig A in S1 Text. Bacterial load in individual worms over time. Data are shown for bacterial species: A) MYb27 (red), B) MYb45 (green), C) MYb53 (blue), D) MYb238 (brown) to illustrate colonization dynamics. Gillespie simulations (blue lines, n=30) were carried out using parameters obtained from fitting CFU per worm data to the mean field model. Mean CFU at each time point (large blue point) and the mean-field deterministic solution (dashed black line) are also shown.Fig B in S1 Text. Fluorescent bead accumulation in adult worms. Worms were exposed to high (20 μL beads per 1 mL buffer) or low (2 μL per 1 mL buffer) concentrations of beads (top labels). Additional nutrient source (heat-killed OP50 or none) is shown on the vertical labels. Samples were taken 10-60 minutes after exposure to beads, and total green fluorescence was measured on BioSorter. Each point represents one individual worm (30-60 worms per sample). Horizontal lines represent 90th percentile GFP in bead-free worms (red, HKOP50; black, no nutrient source) To the extent that the variability in the bead accumulation for the same conditions spans only about an order of magnitude, the intestinal input-output processes cannot account for a large fraction of bacterial load variability in the worms.Fig C in S1 Text. Bacterial load distributions under additional levels of inhibition of growth within hosts. Green fluorescence in MYb14-GFP-KmR pre-colonized worms over 72 hours of inhibition at (a) 100μg/ml or (b) 250μg/ml concentrations of chloramphenicol (CM). Centers and weights of high and low GFP modes at each time point from the transformed GMM fits are shown in the legend. Mean GFP (black dots, dashed lines) for the entire population at each time point is shown. Mean autofluorescence (light green, dashed lines) calculated using the data from uncolonized worms is also shown. (c) Probability density fits modeled as a Gaussian mixture with two components fit on CFU transformed data and then transformed back [file pcbi.1013110.s001.pdf]

S1 Text: Supporting Information

Variance in *C. elegans* gut bacterial load suggests complex host-microbe dynamics

Satya Spandana Boddu, K. Michael Martini, Ilya Nemenman, Nic M. Vega

Logistic growth model fits for additional single species colonization data

As previously described, bacterial load in worms was measured by destructive sampling at 3, 12, 18, 24, 36, 42, and 48 hours after initial colonization. Data for MYb71 and MYb120 are shown in Fig 1. Here, we show the mono-colonization data for the other bacterial species in the minimal microbiome (Materials and Methods: Table 1). We also show the respective Gillespie simulations ($n = 30$) using the parameters (Table A in S1 Text) from mean-field equation fits. These fits are to the means of the data – showing that demographic noise alone is insufficient to explain the variance.

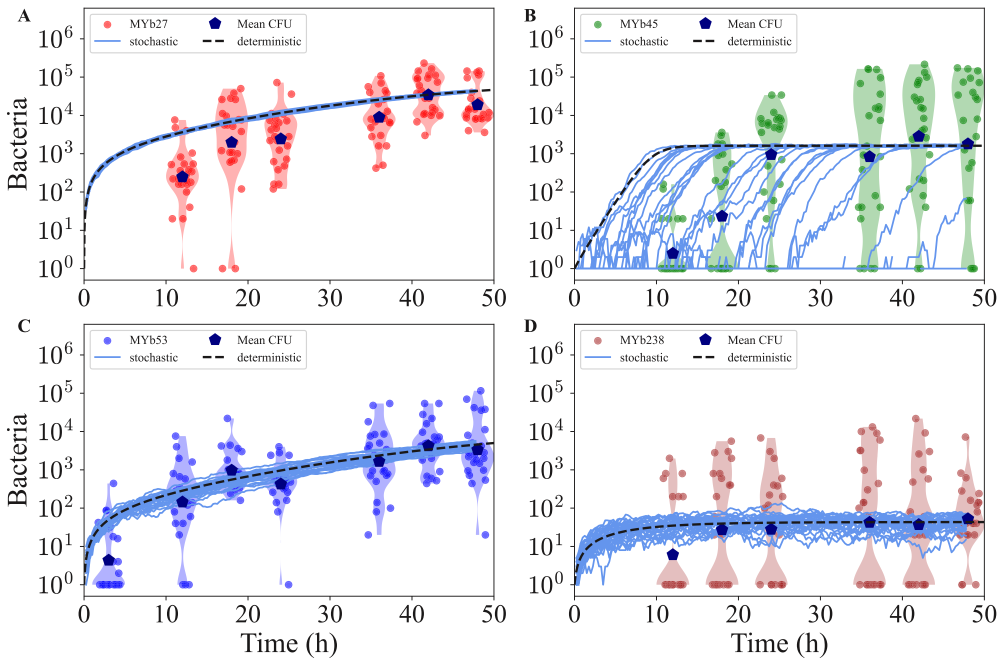

Figure A. Bacterial load in individual worms over time. Data are shown for bacterial species: A) MYb27 (red), B) MYb45 (green), C) MYb53 (blue), D) MYb238 (brown) to illustrate colonization dynamics. Gillespie simulations (blue lines, n=30) were carried out using parameters obtained from fitting CFU per worm data to the mean field model. Mean CFU at each time point (large blue point) and the mean-field deterministic solution (dashed black line) are also shown.

Species	V	c	b	d
MYb27	1.02×10^6	191.70	1.04	0.97
MYb45	5.9×10^4	0.19	2.80	2.04
MYb53	1.38×10^4	14.43	1.07	1.00
MYb71	1.76×10^4	0.07	1.23	0.58
MYb120	1.47×10^2	0.09	2.58	1.40
MYb238	2.60×10^6	5.85	0.85	0.99

Table A. Parameters from logistic model fits to bacterial load data during mono-colonization of N2 worms (Fig 1,Figure A in S1 Text).

Low variability in ingestion and excretion processes

The *C. elegans* intestine is colonized by ingested bacteria that survive transit through the pharyngeal grinder and enter the intestine as live cells. Colonization rate is a function of the density of bacteria in the colonizing inoculum [11] and is an easily-accessible experimental knob in this system, as well as a potential source of variation between individuals. Similarly, the bacteria are lost from the worm gut due to defecation, and the rate of this process contributes to the variability of the bacterial population in the worm. Thus, we sought to verify if the combined variability of these two processes across the individual worms, even in the same external environment, can account for the observed variability of the bacterial density in the worms.

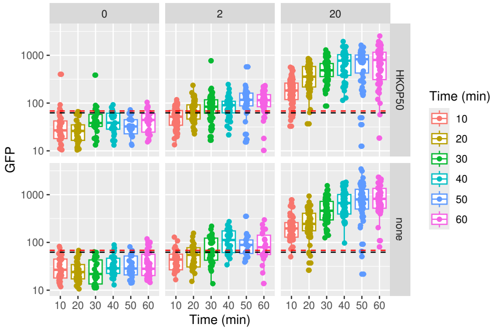

Figure B. Fluorescent bead accumulation in adult worms. Worms were exposed to high (20 μL beads per 1 mL buffer) or low (2 μL per 1 mL buffer) concentrations of beads (top labels). Additional nutrient source (heat-killed OP50 or none) is shown on the vertical labels. Samples were taken 10-60 minutes after exposure to beads, and total green fluorescence was measured on BioSorter. Each point represents one individual worm (30-60 worms per sample). Horizontal lines represent 90th percentile GFP in bead-free worms (red, HKOP50; black, no nutrient source) To the extent that the variability in the bead accumulation for the same conditions spans only about an order of magnitude, the intestinal input-output processes cannot account for a large fraction of bacterial load variability in the worms.

To isolate effects of colonization and excretion from bacterial cell division and death, we investigated these processes using fluorescent beads, whose accumulation processes are similar to those for bacteria [36,47]. To this end, we provided synchronized adult N2 (wild-type) worms with *E. coli* sized fluorescent beads (0.21 μm) suspended in worm buffer and monitored acquisition of beads over time. Beads were provided at a high (20 μL per 1 mL buffer) or low (2 μL) concentration; bead-free worms (0 beads) were used as controls to determine auto-fluorescence thresholds. During bead ingestion, worms

were either provided with heat-killed OP50 ("HKOP50") as an inert but nutritious food source to encourage pumping, or given only the indigestible beads in suspension.

Worms accumulated fluorescent beads over time at an apparently constant mean rate (Figure B in S1 Text). There was much more bead accumulation in the high-bead condition (20 μL/mL) than in the low-bead condition (2 μL/mL), as expected. Across conditions, bead signal in the worm intestine begins to saturate by 40-50 minutes, prior to which there is a linear regime. Further, no-food and heat-killed-food conditions are very similar in overall bead accumulation. This is more consistent with an input-output balance than with the worm gut filling to capacity, and not consistent with the idea that worms can distinguish between different types of particles when feeding in liquid [47]. The observed variation in GFP fluorescence in bead-fed worms covers approximately one order of magnitude, as compared with two to three orders of magnitude observed in bacterial load data, Fig 1, indicating that variation in intestinal input-output processes is not sufficient to account for the observed variation in bacterial load.

Additional conditions for inhibition of bacterial growth in the intestine also show emergence of subpopulations with different bacterial loads

MYb14-GFP-KmR pre-colonized wild-type worms were moved to 100μg/ml and 250μg/ml chloramphenicol, in addition to the conditions previously described in Fig 4 in the main text. These additional inhibition conditions show similar trends as in the conditions shown in the main text, where higher inhibition resulted in more worms in the "low" fluorescence sub-population. This result further indicates the emergence of sub-populations in total bacterial load.

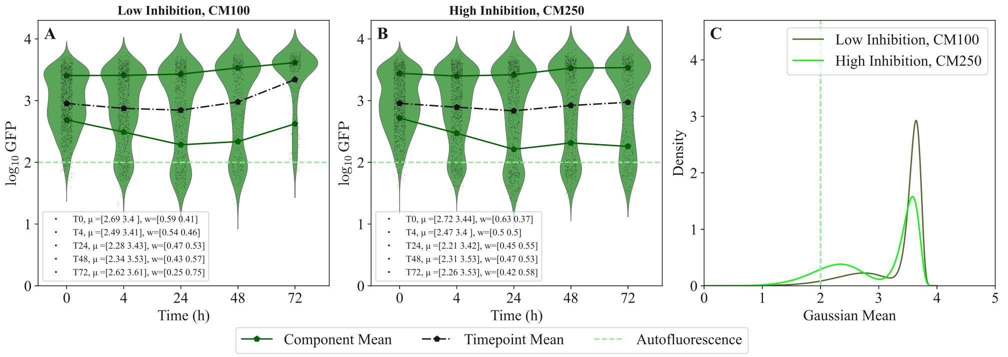

Figure C. Bacterial load distributions under additional levels of inhibition of growth within hosts. Green fluorescence in MYb14-GFP-KmR pre-colonized worms over 72 hours of inhibition at (a) 100μg/ml or (b) 250μg/ml concentrations of chloramphenicol (CM). Centers and weights of high and low GFP modes at each time point from the transformed GMM fits are shown in the legend. Mean GFP (black dots, dashed lines) for the entire population at each time point is shown. Mean autofluorescence (light green, dashed lines) calculated using the data from uncolonized worms is also shown. (c) Probability density fits modeled as a Gaussian mixture with two components fit on CFU transformed data and then transformed back to GFP, at T72 for CM100 and CM250 inhibition.

Bacterial load switched between two modes similarly in different experimental runs

To establish that our results on emergence of multiple bacterial load subpopulations are robust, we repeated experiments in Fig 5 multiple times. Specifically, wild type worms were colonized with MYb14-GFP-KmR for 24 hours before being separated into high and low bins, as described before. Green fluorescence was measured 24 and 48 hours post separation. The experimental results were always similar to that of Fig 5 in the main text, but in some experiments, Figure Din S1 Text, there was higher handling-related loss of worms by 48 hours, resulting in smaller worm populations. Despite the smaller number of worms, we see similar trends, with re-distribution of individual worms between apparent states in total bacterial load as measured by GFP fluorescence.

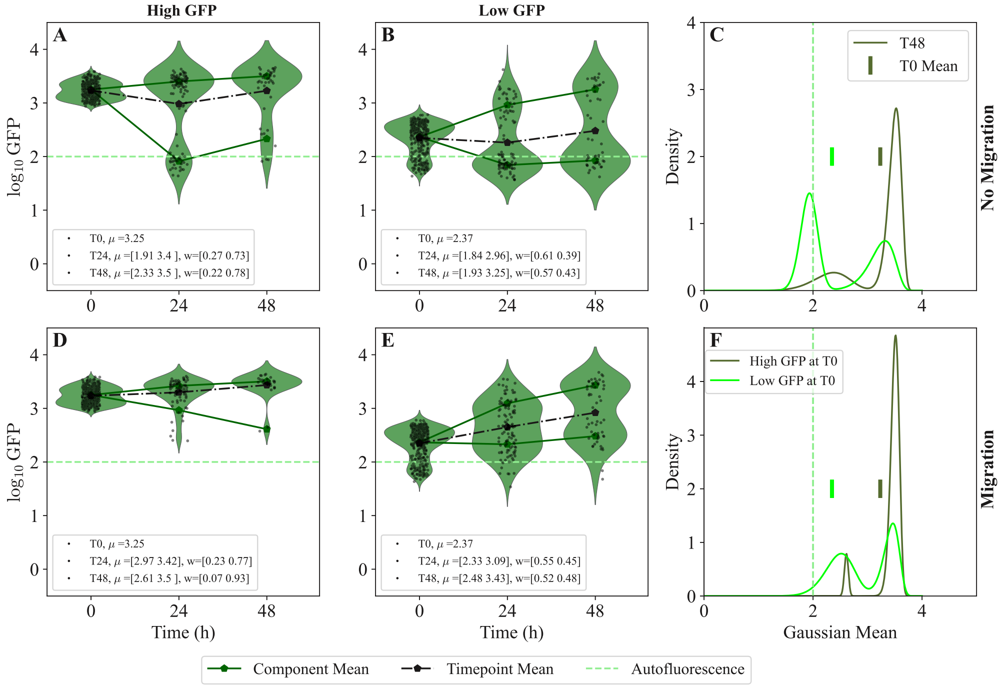

Figure D. Bacterial load switching between modes in an additional experimental run. Green fluorescence over 48 hours after MYb14-GFP-KmR pre-colonized worms were separated based on high (A,E) and low (B,F) GFP under conditions of A-C) No Migration (top) or E-F) Migration (bottom). Mean GFP (black dots, dashed lines) for the entire population at each time point is shown. Mean auto-fluorescence (light green, dashed lines) calculated using the data from uncolonized worms is also shown. C,F) PDFs using transformed GMM fits at T48 for worms starting at low (light green) and high (dark green) fluorescence in no migration (top) and migration (bottom) conditions.

State switching is also observed with other bacteria

Although the focus of our experiments was on the commensal MYb14-GFP, state switching in total bacterial load was also observed in worms colonized with other bacterial species. For example, in an unrelated experiment [21], N2 worms colonized

with GFP labeled *Salmonella enterica* LT2 were separated into high- and low-fluorescence bins, surface bleached, and transferred individually into wells of a 96-well plate containing S medium + heat-killed OP50 + kanamycin for selection. In parallel, a sample of worms from each gate was taken for measurement of bacterial load in individual worms (see Materials and Methods: Single Species Colonization). After 48 hours, worms were retrieved from wells, and bacterial load was measured from these individuals. We observed that, as with MYb14-colonized worms, distributions of bacterial load shifted over time, with some low-GFP individuals transitioning to a highly-colonized state and vice versa, Figure E in S1 Text.

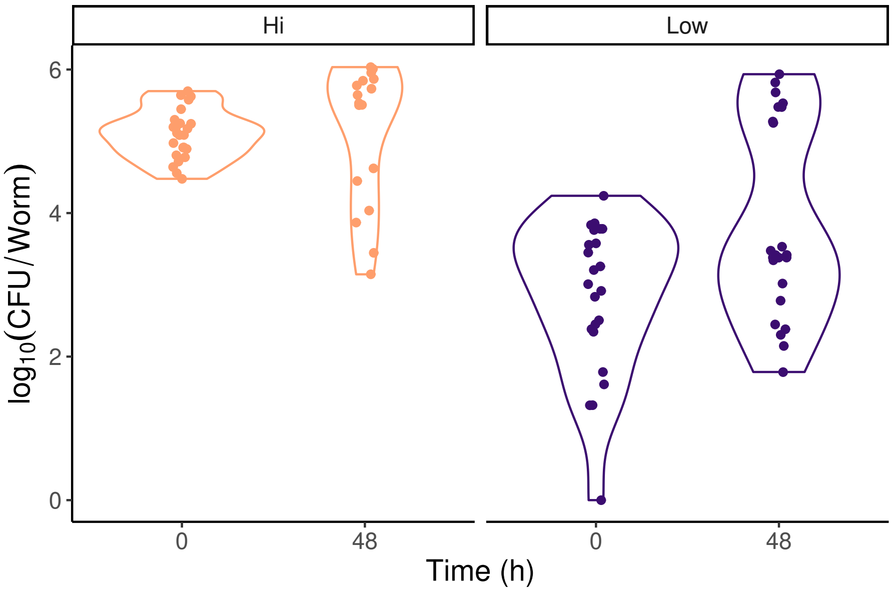

Figure E. State switching in N2 adults colonized with *Salmonella enterica* LT2. Adult worms were allowed to feed on lawns of *S. enterica*-GFP for 48 hours, then purged and sorted into bins based on GFP fluorescence as before. Individual worms were distributed into wells of a 96-well plate containing S medium + 1X heat-killed OP50 + 50 μg/mL each kanamycin (for selection) and chloramphenicol (to prevent re-inoculation) for 48 hours. Individual worms ($n = 24$) were digested at the start (time 0) and end (time 48) of the experiment.

Sample fits of models of bacterial load to GFP measurements

Figure F in S1 Text Panel A shows an example fit of a GMM of two components to data from Fig 4 at 24 hours, fitted to log(CFU) data transformed using Eq. 7. Panel B shows the same data in log(GFP) space and the pdf of the transformed GMM model using Eq. 8. Tables Table B and Table C show the means, standard deviations, and weights of a two component GMM model fit to transformed log(CFU) data for multiple experimental conditions described in the *Main text.*

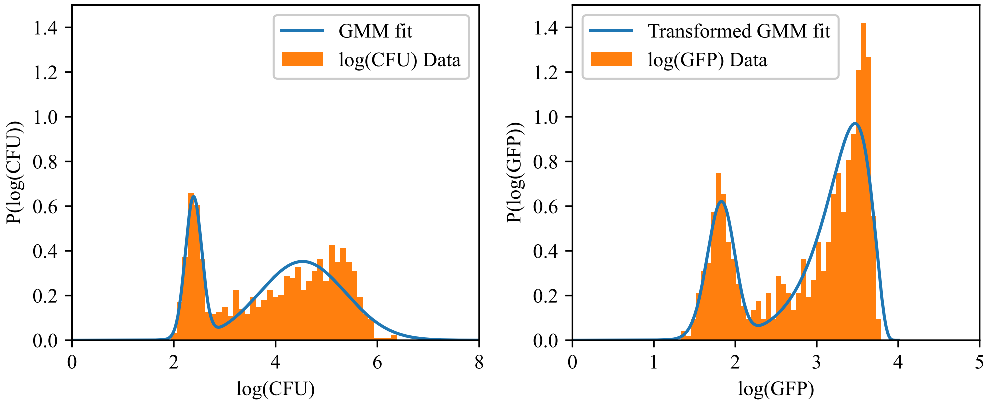

Figure F. Probability distributions of GFP and CFU data. Left: log(CFU) probability distribution using transformed log(GFP) from Fig 4 at 24 hours. GMM with two components is fitted to the log(CFU) data. Right: The GMM distribution fitted in the log(CFU) space is transformed back to the log(GFP) space.

Condition	Time (h)	Mean 1	Mean 2	Std 1	Std 2	Weight 1	Weight 2
CM25	0	3.40	4.76	0.47	0.49	0.62	0.38
CM25	4	3.01	4.66	0.53	0.55	0.49	0.51
CM25	24	2.39	4.53	0.16	0.84	0.26	0.74
CM25	48	2.90	5.33	0.37	0.47	0.25	0.75
CM25	72	3.46	5.56	0.56	0.40	0.22	0.78
CM500	0	3.32	4.69	0.48	0.53	0.57	0.43
CM500	4	3.08	4.69	0.54	0.55	0.51	0.49
CM500	24	2.87	4.69	0.56	0.56	0.47	0.53
CM500	48	2.88	4.95	0.54	0.51	0.46	0.54
CM500	72	2.75	4.89	0.43	0.57	0.44	0.56

Table B. Raw parameters from the GMM in the log(CFU) space fitted to data in Fig 4.

Modeling in the log(CFU) space results in a multiplicative noise process

As described in the previous section, we chose to model our experiments in the log(CFU) space, which we consider to be more easily related to real bacterial loads. Additionally, the fluctuations in log(CFU) space are more symmetric, making fitting models easier.

The question then arises what the equivalent model in the CFU (not logarithmic) space is. This is answered by performing a stochastic change of variables using Ito's lemma. Equation 4 of the main text undergoes a transformation under the change of variables $w = exp(\phi)$ becoming:

$$\frac{\partial w}{\partial t} = f\left(\ln(w)\right) \cdot w + \frac{Dw}{2} + \sqrt{D} w \eta, \tag{1}$$

where the $Dw/2$ is a drift term and η is a Gaussian white noise. This transformed equation has multiplicative noise and, as long as f is a polynomial, it prohibits any kind of pure migration term in the traditional sense of a constant growth rate. For experiments where a traditional migration term is expected either the data should be fit in non-log transformed coordinates or f should contain a decaying exponential as one of its terms.

Condition	Time (h)	Mean 1	Mean 2	Std 1	Std 2	Weight 1	Weight 2
High -	0	4.36	4.36	0.40	0.40	1.00	1.00
High -	24	2.41	4.82	0.15	0.34	0.48	0.52
High -	48	2.78	4.96	0.33	0.42	0.44	0.56
Low -	0	3.09	3.09	0.32	0.32	1.00	1.00
Low -	24	2.37	3.82	0.11	0.54	0.71	0.29
Low -	48	2.41	4.47	0.17	0.51	0.68	0.32
High +	0	4.32	4.32	0.41	0.41	1.00	1.00
High +	24	3.57	4.89	0.41	0.26	0.34	0.66
High +	48	3.27	5.00	0.84	0.33	0.29	0.71
Low +	0	3.14	3.14	0.28	0.28	1.00	1.00
Low +	24	3.23	4.41	0.45	0.49	0.63	0.37
Low +	48	3.59	4.97	0.48	0.28	0.61	0.39

Table C. Raw parameters from the GMM in the log(CFU) space fitted to data in Fig 5.

Mathematical models struggle to predict quantitative details of the dynamics of the bacterial load

The mathematical models considered here fit the distributions of the bacterial load data with quantitative precision. Here we verify if these models can predict the dynamics of the load as well. For this, in Figure G and Figure H in S1 Text, the dynamics of the bacterial load is simulated using both the potential model and the switching model and predictions of simulations are compared to the GMM fits to the data at the future time points. The parameters for the GMM and the potential models were fit directly to data and the parameters for the switching model were inferred from the means, variances, and weights of the corresponding GMM fit, as described in Materials and Methods: Probability distributions of bacterial load in different mathematical models. For this fit, we used the 24 hour time point in the low inhibition experiment of Fig 4. This time point is 72 hours after the *C. elegans* were initially colonized and is, therefore, on the same day of adulthood as the 48 hour time point of the no migration condition of Fig 5. In this context, the main difference between these two time points is that in Fig 5 the population is filtered to separate *C. elegans* with high or low populations of bacteria. This allows us to compare the observed re-distribution of individuals across states in Fig 5 with predictions from simulations initialized using the same distributions of individuals.

In both simulations, the low sub-population quickly recovers to the original distribution that was fit. The simulations reach steady state at a faster time scale than observed in the experimental data. The simulations do, however, capture the means of the modes and the qualitative features of the relaxation dynamics observed in the experiments. Larger amounts of data are needed to fit the state switching dynamics in either of the two models considered here. For example, in the state switching model, one would be able to question the assumption of memoryless switching, and, in the potential model, one could introduce latent states in the system or consider even higher order potentials.

These results illustrate the main distinction between the state switching and the potential models: they predict somewhat different dynamics of the bacterial loads. For the state switching model, the rates of switching are parameters, which can be tuned independently of the curvatures of the potentials and hence of the variance of populations in each of the states. For the multistable potential model, the curvatures of the potential (and hence the variance of the fluctuations) contribute to controlling the

switching rate. Thus, collecting longitudinal data, measuring the switching rates, and observing its relationship to the fluctuations about a state would be one way to distinguish between these models.

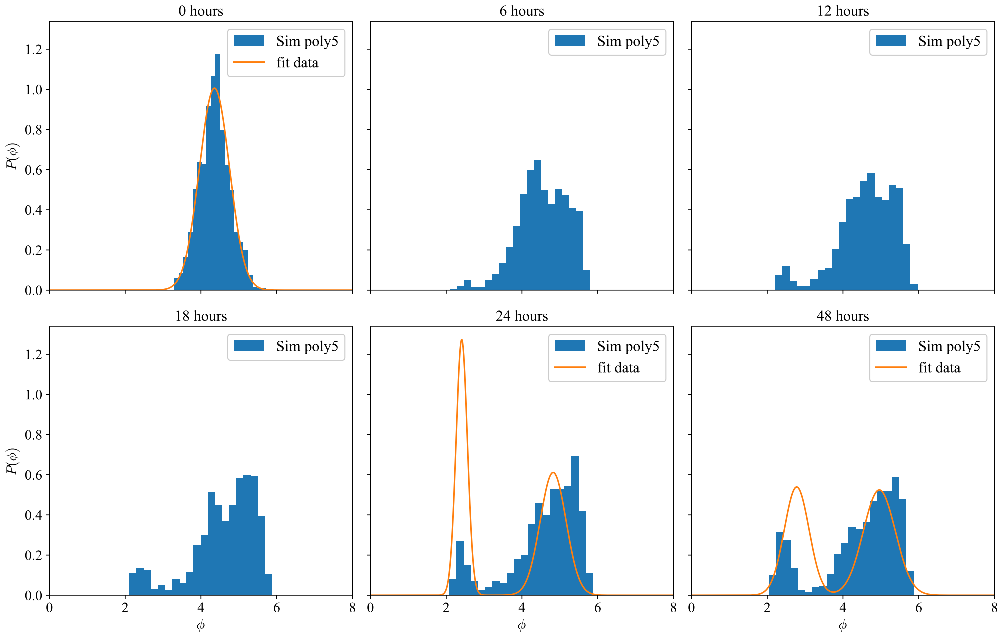

Figure G. Predictions of dynamics in the 6th order polynomial potential (5th order force) model. We fit the potential to data in Fig 4 at 24 hours. We start then with the initial distribution of bacteria from the High GFP condition of Fig 5 (top row) (shown as a histogram in the top left panel here), and fit it to a GMM (orange line in the top left panel). We evolve this distribution according to the dynamics in Materials and Methods: Probability distributions in the potential model (histograms in all other panels), and compare to the GMM fits to 24 and 48 hour data in Fig 5 (shown here as orange lines in the last two panels). Times 6,12, and 18 are from simulation alone are for visualization purposes only, we do not have the corresponding data for these times.

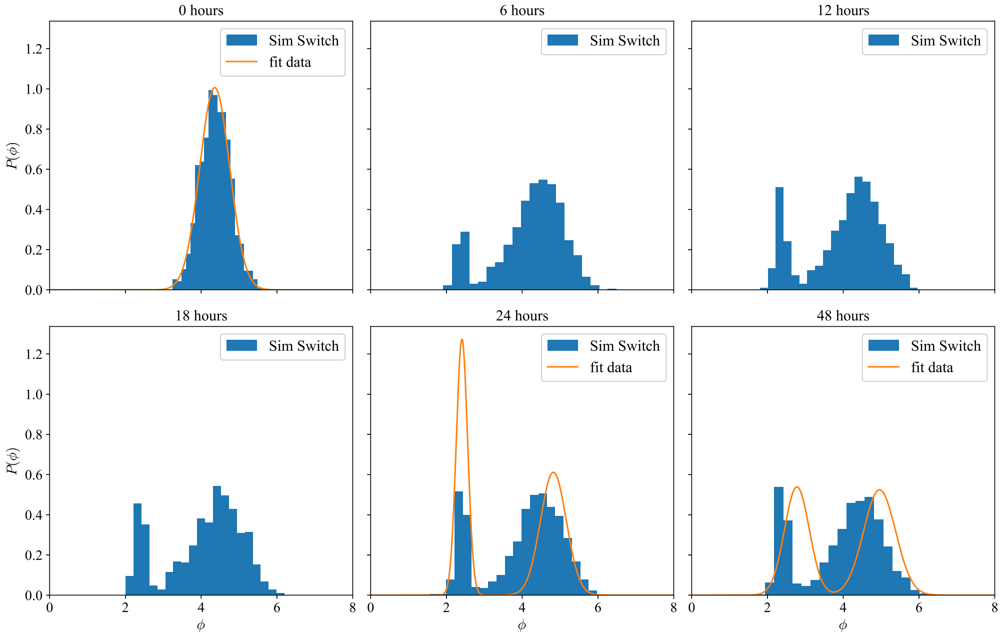

Figure H. Predictions of dynamics in the state switching model. Plotting conventions are the same as in Figure G in S1 Text, except that the fits and the dynamics are now done using the state switching model. Times 6,12, and 18 are from simulation alone are for visualization purposes only, we do not have the corresponding data for these times.
