# Supplementary material for: Variance in C. elegans gut bacterial load suggests complex host-microbe dynamics
Source: PLoS Comput Biol. 2025 Jun 9;21(6):e1013110. doi: 10.1371/journal.pcbi.1013110 (PMC12180659; doi:10.1371/journal.pcbi.1013110)
Supplement: Attachment — Submitted filename: ResponseLetter.pdf [file pcbi.1013110.s002.pdf]

400 Dowman Dr.
Emory University
Atlanta, GA 30322

April 28, 2025

Dr. Samir Suweis
Academic Editor
PLOS Computational Biology

Re: PCOMPBIOL-D-25-00094
Variance in C. elegans gut bacterial load suggests complex host-microbe dynamics
PLOS Computational Biology

We thank the Editor and the Reviewers for their time and comments. We appreciate that both Reviewers responded favorably to our manuscript and found that our manuscript is well written and well suited for PLOS Computational Biology. We have responded to both Reviewers' comments and questions in the attached letter.

We used red color for quoting directly from the Referees and the Editor, and black for our responses. Additionally, we used blue color to highlight changes we made, along with the corresponding line numbers in the manuscript, in response to the Referees. A marked `latexdiff` file that shows the changes to our manuscript is also attached with this submission.

The following is our response to each point raised by the reviewers.

Sincerely,
Satya Spandana Boddu
K. Michael Martini
Ilya Nemenman
Nic Vega

Additional Editor Comments:

Both reviewers find your work very valuable and well written. They propose some suggestions and comments that I invite the authors to consider to further improve their work.

Thank you for this positive feedback!

Reviewers' comments:

Reviewer #1:

The study investigates variability in C. elegans gut bacterial load and proposes that this variability cannot be explained by demographic noise or static host heterogeneity alone. Through a combination of experiments and mathematical modeling, the authors suggest that the host-microbe system switches stochastically between high- and low-growth phenotypes.

The article is quite clear and presents results that, to the best of my knowledge, are original and are undoubtedly on par with Plos Computational Biology. I have questions about the part about the model, particularly with respect to the mechanisms that were decided to be incorporated.

We thank the Reviewer for their favorable evaluation of our manuscript and their recognition of the importance of understanding the variability of *C. elegans* gut bacterial load. We answer the questions below.

1) Why is a white noise model chosen instead of considering a colored noise process or state-dependent fluctuations? Is it merely a decision of convenience, or is there a clear biological motivation?

We discussed this question in depth in the supplementary material, specifically in Sections "Calibration of log(CFU) to log(GFP) mapping" and "Modeling in the log(CFU) space results in a multiplicative noise process". In "Modeling in the log(CFU) space results in a multiplicative noise process" we note the following regarding this question:

> ..., we chose to model our experiments in the log(CFU) space,

> which we consider to be more easily related to real bacterial loads. Additionally, the fluctuations in log(CFU) space are more symmetric, making fitting models easier.
> The question then arises what the equivalent model in the CFU (not logarithmic) space is. This is answered by performing a stochastic change of variables using Ito's lemma. Equation 4 of the main text undergoes a transformation under the change of variables $w = exp(\phi)$ becoming:
>
> $$\frac{\partial w}{\partial t} = f\left(\ln(w)\right) \cdot w + \frac{Dw}{2} + \sqrt{D}w\eta, \qquad (1)$$
>
> where the $Dw/2$ is a drift term and η is a Gaussian white noise. This transformed equation has multiplicative noise and, as long as f is a polynomial, it prohibits any kind of pure migration term in the traditional sense of a constant growth rate. For experiments where a traditional migration term is expected either the data should be fit in non-log transformed coordinates or f should contain a decaying exponential as one of its terms.

That is to say, we do actually use multiplicative noise in CFU space. We chose to use white noise in log(CFU) space because the fluctuations appear roughly symmetric and Gaussian when log(GFP) is transformed to log(CFU).

2) It appears that the authors argue that the observed variability in bacterial load within C. elegans does not require microbial interactions to explain, as even when the worms were mono-colonized (i.e., exposed to a single bacterial species), a large variability in bacterial load was still observed. However, they do not delve into possible mechanisms by which the absence of microbial interactions is not a relevant factor. For example, they do not rule out that previous microbial interactions or environmental factors may have influenced the observed dynamics. Similarly, they do not explore whether the variability could be better explained in a context of competition or cooperation between bacteria rather than just considering stochastic state transitions. Was the possibility of past or indirect microbial interactions influencing the observed variability evaluated, even under mono-colonization conditions? Were models of competition or cooperation between bacteria explicitly ruled out?

We do not rule out that bacteria interactions can impact the fluctuations in natural microbiomes. However, we do show that, in mono-colonized *C.*

elegans, density fluctuations are still high, meaning that interactions between bacterial species are not necessary to explain these fluctuations.

This is not to say that some of the natural variation in whole microbiomes cannot come from interactions among microbial species. Yet, our results can be interpreted as saying that this won't be enough and that that either interactions between bacteria of the same species or between bacteria and their host are also necessary. We proposed two models, each of which could emerge from multiple specific, biological mechanisms: 1) a state dependent switching model modulated by the environment, and 2) a phenomenological model where the density-dependent potential could be due to intra-species interactions between bacteria. We now clarify our interpretation of the potential models to include the fact that these models are agnostic as to the specifics of the underlying mechanism(s), including the possibility that the density dependence of the potential could indicate interactions between bacteria of the same species. (Lines 387-389)

Likewise, as these worms were sterile at the point of mono-colonization, inter-species bacterial interactions are not present; however, worms were raised on *E. coli* as a food source, and it is always possible that rearing conditions will differentially affect the physiology and/or behavior of the host. We have not extensively investigated the effects of different rearing conditions on microbial load dynamics. However, it is worth mentioning in this context that wide variability in microbial load is observed across a substantial range of experimental conditions (different host genotypes; feeding on plates vs in liquid; different rearing temperatures; different single-species colonists; microbial communities) (this manuscript and https://www.biorxiv.org/content/10.1101/2022.07.25.501406v3) - in short, it is very difficult not to observe this property of the worm-microbe system. We have added this material to the Discussion. (Lines 501-508)

Once the authors addressed, at least qualitatively, the two issue above, the manuscript will be suitable for pubblication in Plos Computational Biology.

We hope that the explanations we provided will be sufficient to alleviate the concerns raised.

Reviewer #2:

This paper addresses the important question of the in vivo colonization of the gut by microbes. The approach taken of focusing on one bacterial species in a host which presents as best as possible reproducible conditions is very relevant to understand this complex problem. The authors showed in an extensive manner that the logistic model could not explained their data. They propose two models that can reproduce the data and suggest experiments which should be able to discriminate between the two models. I recommend this paper for publication.

We thank the Reviewer for their positive evaluation of the paper and their helpful comments and questions that we address below.

Comments

- Line 78: Could you explain why what would be expected for σ/μ from demographic noise? It does not seem obvious to me from [23] that the linearity between mean and standard deviation exclude the demographic noise as the source of variability.

Upon revisiting this statement, we agree that the framing is unnecessarily confusing. Furthermore, the reference is more directly relevant for Taylor's law relationships in time series, which is not the data type we are using. We have thus removed the reference. Qualitatively, we expect that with demographic noise, relative variation should decrease as the system size increases.

- Line 81: Could you be more specific about the long-tailed distribution and fit your experimental data with the specific distribution which is expected?

We don't have an expectation for our experimental data, and so we cannot be more specific here. We clarified this in the text. (Line 80) By making this statement, we wanted to draw attention to the fact that our data is long-tailed and skewed, similar to previous experimental results (see Figure 1 in the reference).

- Very nice Figure 5!

Thank you!

- For model 1, could you explain the shape of $f(\phi)$ with biological arguments?
We really don't know. However, if pressed to answer, we guess that this

can be explained by a population density-dependent birth rates or death rates. These rates themselves can come from interactions between bacteria of the same species. We have added this speculation to the main text. (Lines 424-426)

- For model 2, it would be interesting to propose hypotheses as to why they are two states. What would be the biological origin of those two different states? What would trigger the change from one to the other state?

For this model, the two states could be something as simple as whether the *C. elegans* is feeding or not. It could also be some some physical change in the gut, the mucosa, or the spatial structure of the bacteria in the gut. We have also added this speculation to the main text.(Lines 433-435)

- Could it help discriminating between the model to add "fit data" at intermediates times (6 hours, 12 hours, 18hours) in Fig 16 and 17? From Fig1 for instance it seems you do have access to experimental data to do so.

Here we fitted high throughput GFP histograms of the distributions of bacteria loading. We don't have the corresponding GFP data at these intermediate times (6, 12 and 18 hours) for the later experiments (Figure 4 and 5). What is shown in Figure 16 and 17 were predictions of the polynomial and state-switching models and are not actual fits to the data. We have clarified in the supplementary information (Fig. 16 and Fig. 17 captions) that these intermediate times are from simulations.

- Page 15: Line 454: Clearly, as the order of the potential model is increased it becomes better able to fit the empirical bacterial load distribution.
Could you comment on the fact that you are not actually overfitting? (also for the state switching model)

It is possible that we are overfitting, but overfitting is much less likely when we are using low order polynomials. Qualitatively, we clearly see two modes of bacterial density, which requires at the minimum a 4th order polynomial for the potential to have two stable population densities. Higher order polynomials are more likely to be an overfit.

Similarly, in order to produce two stable population densities in the switching model, at least the carrying capacities need to be modulated. And additional parameters are needed when one tries to fit densities at intermediate bacterial loads.

Minor comments

Page 1: In the author's summary, I think the sentence is difficult to understand (especially before having read the paper): We found that bacteria behaved differently when grown in a host compared to the standard logistic growth observed in vitro, exhibiting population density-dependent growth or the emergence of two primary subpopulations of bacteria. I would reformulate.

We agree and we have rephrased the author's summary to be clearer.

Page 2: We found that high variation is still present in bacterial load of mono-colonized worms, indicating that microbe-microbe interactions are not required to produce inter-individual differences in microbial colonization of the worm intestine. This sentence suggests that microbe-microbe interactions will produce inter-individual differences. Is it possible that those interactions will decrease them? (by constraining the dynamical behavior for instance, or some sort of self-organisation).

To be clear, we meant microbe-microbe of different species interactions. We have clarified this in the main text. (Line 45) It could be that there are still interaction between microbes of the same species, and in fact we find this is likely later on in the manuscript. It is true that microbe interactions between species could either increase or decrease the fluctuations, but we don't know this for sure. That said, we would expect it to be more likely for the variation to increase due to additional interactions as independent sources of variation tend to add additively.

Page 2: Instead, we found emergence of apparent alternative states in bacterial load inside worms, with movement of individuals between these states, suggesting that canonical models of bacterial growth cannot fully characterize the host-microbe system's dynamics. I would use the word transitions rather than movement.

We have made this change to the text to make it clearer. (Line 50)

Page 2 last paragraph: I find it confusing that you write that the two models cannot be distinguished based on snapshots data while you do have time series available.

What we mean by time-series long longitudinal (not an individual snapshot) data from the same worm. The data that we have is collective (population measurements), which doesn't not allow for observing trajectories of individuals. Additionally, the data is of a snapshot nature, so that we cannot follow the same worm. We have made this point more explicit in the main

text. (Lines 53-55)

Page 4: In Fig1 B (and C), how could the deterministic curve to on top of simulations? The deterministic curve should be the average of all simulations. Maybe my impression from the figure is wrong because of the log scale. The figures B and C are convincing, but it could be easier to have violin plots at the time points where the experimental data is taken for the theoretical model to compare the variance in the experimental and theoretical cases. (Maybe around t=10h for instance, the variance is well captured by the model?)

In our model, we consider the well-mixed volume inside the worm and the carrying capacity of the worm to be the same. This results in a smaller fluctuation amplitude when compared to a smaller well-mixed volume size scenario. In any case, the fluctuations should be evenly distributed on the both sides of the mean. In C) this is clear for later times as there are minimal number of transient simulations. It is misleading in case of B) as most of the simulations are in transient state. We have edited the text to make this point clear. (Lines 101-105)

Page 5: to help the reader, I would add the name of the parameters after ingestion and excretion rates on line 117. Is it correct that those parameters are named as follow: bi = birth parameter, ci colonization/ingestion parameter, di excretion/death?

It is correct. We agree that adding the parameter name would help the reader and have made the corresponding change to clarify what these parameters mean. (Lines 123-124)

Page 16: You could cite https://doi.org/10.7554/eLife.55650 with ref [39]. The papers reached the same conclusions with different approaches.

We now also cite this paper. (Line 531)

Page 24: Fig. 10: it is not obvious to me that the fits are good (in particular for panel C). My impression is maybe due to the log scale. Could you provide a quantitative assessment of the quality of the fit?

These fits are just to the mean and do not capture the full variance solidifying our point that demographic noise is not sufficient. We have added additional text in the main text and supplemental information (Line 8) to make this clear.

Page 31-32: could be nice to combine figures 16 and 17 to be able to compare more easily.

Thanks for the suggestion. However, by doing so, we believe it would be confusing to distinguish between distributions.

Rem: The supporting information is not well structured. Could you remove the “1” in the title page 27 and “2” page 28? Or maybe start with a “1” on page 24 for “Logistic growth model fits . . . ” ?

We have removed the section numbers and used just the titles instead. Thank you for pointing out this inconsistency.

Sincerely,
Satya Spandana Boddu
K. Michael Martini
Ilya Nemenman
Nic Vega
